# Evaluation of the food effect on a drospirenone only contraceptive containing 4 mg administered with and without high-fat breakfast in a randomised trial

**DOI:** 10.1186/s12905-022-01960-2

**Published:** 2022-09-19

**Authors:** P.-A. Regidor, W. H. Richter, R. Koytchev, V. Kirkov, E. Colli

**Affiliations:** 1 Exeltis Healthcare, Adalperostr. 84, 85737 Ismaning, Germany; 2 CCDRD AG, Lindenallee 70, 15366 Hoppegarten, Germany; 3 MHAT Tokuda Hospital, Sofia, Bulgaria

**Keywords:** Drospirenone, Bioavailability, Pharmacokinetic, Food intake

## Abstract

**Background:**

The objective of the present trial was to assess the difference in pharmacokinetics (PK) of an oral test preparation containing 4 mg drospirenone (DRSP) under fasting conditions compared to PK upon food intake after single dose administration.

**Methods:**

Open label, single centre, two-treatment, two-sequence, crossover study in 24 healthy female volunteers, with duration of 1 day per sequence and with a real wash-out period of 14 days to investigate the relative bioavailability of DRSP with both forms of administration. The 90% confidence intervals (CI) were calculated for the intra-individual ratio (test with food vs. without food) of the PK endpoints Area under the curve; 0–72 h [AUC(0-72 h_)_] and maximal plasma concentration [Cmax] of DRSP.

**Results:**

The 90% CI calculated by analysis of variance using logistic transformation (ANOVA-log) for the endpoint, intra-individual ratio (Test ‘A’ = with food intake) vs. Test ‘B’ = without food intake) of AUC(0-72 h) of drospirenone was between 104.72 and 111.36%. The 90% CI calculated by means of ANOVA- log for the endpoint intra-individual ratio (Test ‘A’ vs. Test ‘B’) of Cmax of DRSP was between 118.58 and 141.10%. The mean relative bioavailability of the test with food ‘A’ compared to the Test without food ‘B’ after single dose administration based on the endpoints AUC(0-72 h_)_ was 107.99%; for the endpoint Cmax it was 129.35%.

**Conclusions:**

The rate of absorption, based on the endpoint Cmax of DRSP was increased by about 30% under fed conditions. With respect to consumer habits, this may represent a relevant benefit for contraceptive safety, as the time span between food consumption and pill intake does not play a role.

**Implications:**

Our results suggest that the food intake has no impact on the absorption of 4 mg DRSP in the management of contraception. This increases the contraceptive efficacy as no interference with food is expected when consuming the oral formulation under real life conditions.

*Trail registration*: Trial registration number: EudraCT-No: 2012–004,309-28.

## Introduction

Estrogen-free pills are safe regarding cardiovascular diseases as they do not increase the risk of thromboembolic or stroke events in comparison to contraceptives containing estrogens [1, 2]. Traditional progestin-only pills (POPs) are associated with an unpredictable bleeding pattern, and stringent daily timing and missed pill rules that might affect contraceptive reliability. A new generation of estrogen-free pill containing 4 mg of drospirenone (DRSP) has been developed to improve these aspects.

DRSP is rapidly absorbed following oral administration. Serum DRSP concentrations are linearly related to the amount of DRSP in a single oral dose with a mean bioavailability between 76 and 85%. Nearly 98.5% of circulating DRSP is bound to serum proteins but does not bind sexual hormone binding globulin (SHBG) or corticosteroid-binding globulin (CBG) [3–5]. This contrasts with other progestins such as norethindrone, levonorgestrel, desogestrel and gestodene, which all have binding affinities to SHBG, resulting in less availability of SHBG for androgen binding [6].

DRSP binds to aldosterone receptors in the kidney, blocking the effects of aldosterone and resulting in moderately increased sodium and water excretion leading for example to a reduction in the blood pressure, especially in women with mild hypertension [5, 7–11]. DRSP also binds the androgen receptor (AR) in peripheral tissues, blocking the effects of testosterone [5, 7, 8, 10].

Data from a human mass balance study indicate that DRSP is extensively metabolized, as only trace amounts of DRSP were excreted unchanged in urine and feces. In human plasma, the two major metabolites of DRSP that have been identified are the acid form of DRSP generated by opening of the lactone ring and the 4,5-dihydro-drospirenone-3-sulfate form [12]. Both metabolites are formed independent of the cytochrome P450 (CYP) enzymatic systems [12]. They are considered pharmacologically inactive and are excreted in urine or feces, with almost complete excretion occurring 10 days after administration of single and multiple dose regimens. Plasma levels decline biophysically with a plasma distribution phase half-life of 2 h and a terminal disposition half-life of 30–34 h [3]. Only a minor part of the DRSP metabolism is associated with CYP3A4 and other CYP enzymes [13]. Therefore, CYP3A4 inhibitors should have no significant effect on the pharmacokinetics (PK).

Anyway, some studies reported a PK interaction between CYP3A4 inhibitors and DRSP: co-administration of boceprevir (Victrelis®, Merck Sharp & Dohme), a protease inhibitor used to treat chronic hepatitis C, with a combined oral contraceptive (COC) containing DRSP and ethinyl estradiol (EE) (Yaz®, Bayer) resulted in a twofold increase in DRSP exposure [14]. A subsequent study investigated the potential interaction between the potent CYP3A4 inhibitor ketoconazole and the above-mentioned COC and found a 2.68-fold increase of DRSP exposure in the DRSP with EE group when ketoconazole was co-administered [12].

Interestingly, a comparative PK crossover study with a DRSP-only preparation and the COC containing 3 mg DRSP and 20 µg EE revealed that DRSP administered alone exhibited a lower accumulation ratio than when coadministered with EE. The extent of systemic exposure at steady-state was about 32% less with the DRSP-only formulation (AUC(0-24 h_)_, steady-state geometric mean ratio: 77.8%; 90% confidence interval: 74.6%–81.1%). These results suggest that metabolic pathways of DRSP can be inhibited by EE resulting in higher DRSP plasma concentrations in DRSP/EE formulations than in a DRSP-only formulation and that the enzymes CYP3A4 and sulfotransferase 1A1 (SULT1A1) may play a role in this context [15].

The main objective of the present trial was to assess the effect of food intake on the PK of an oral contraceptive containing 4 mg of non-micronized DRSP after single dose administration. For that, PK under fasting conditions was compared with that observed, when pill intake occurred 30 min after a high-fat breakfast.

## Materials and methods

### Study design

We conducted an open-label, controlled, crossover, 2-treatment, 2-period, 2-sequence, monocentre study at the Sector for Bioequivalence Trials at MHAT Tokuda Hospital, (Sofia, Bulgaria) between January and February 2013 (EudraCT-No: 2012–004,309-28. In one study period, PK under fasting conditions was analyzed, while the other study period investigated the PK under fed conditions. Each of the two study periods lasted 5 days and included two hospitalizations of approximately 24 h (days 0 to 1) and 4 outpatient visits. There was washout phase of 14 days between the two study phases.

### Ethical conduct, study approval and timelines

We performed the study in accordance with Good Clinical Practice (GCP), local requirements and the Declaration of Helsinki. The Bulgarian Drug Agency and the local ethics committee at MHAT Tokuda Hospital (Sofia, Bulgaria) approved the study, and all subjects gave written informed consent.

#### Studied period

Date of first enrolment: 15-JAN-2013 date of first subject dosed: 20-JAN-2013.

Date of last completion: 06-FEB-2013 (last regular final visit), on 22-FEB-2013, additional control visit in 1 subject.

The study is registered in the Eudra Ct clinical registry with the number EudraCT-No: 2012–004,309-28. https://www.clinicaltrialsregister.eu ".

The posted result-related information is made public through the EU Clinical Trials Register of Eudra Pharm in accordance with the Commission guidance documents set out under Sect. 1, i.e., only result-related information on non-paediatric Phase-I clinical trials is not made public.

### Sample size

We calculated the sample size based on residual variance data for the area under the concentration/time curve (AUC) (15%) and the observed maximal concentration (Cmax) (20%) obtained in a preceding pilot PK study. Twenty-four (24) subjects completed the study, which was considered to provide at least 80% power at 5% alpha for an equivalence test with a geometric mean ratio of up to 1.05 and the corresponding confidence interval (CI) within the limits of 80–125%.

### Subjects and treatments

#### Subjects

We conducted the study in pre-menopausal Caucasian women, aged between 18 and 40 years, with a body mass index of ≥ 18.5 to ≤ 30 kg/m^2^. The women were required to be physically and mentally healthy based on medical and standard laboratory examinations, non-smokers since at least 6 months (confirmed by urine cotinine test) and had to be using an effective non-hormonal method of contraception. 24 healthy pre-menopausal female volunteers were randomized and completed both study periods according to the study protocol.

Each of the participants were randomised to one of the two possible study sequences in a way, that each sequence occurred with the same incidence.

#### Treatment

The study drug was administered by the investigator on two single occasions either under fasting conditions in one study period or under fed conditions in the other study period. For that, dosing was performed in the morning of day 1 between 8:00 and 8:46 a.m. after at least 10 h overnight fasting, or 30 min after the start of a standard high-fat breakfast, after checking for exclusion criteria, diet, restrictions, and adverse events. The participants had to remain in an upright position (walking, sitting, standing) for 4 h after administration of the drug. A second medical professional supervised the intake.

Each study phase lasted 5 days and included a hospitalization for 25 h (day 0 to 1) and four ambulant visits at days 2–4 after dosing for blood sampling, and checking for exclusion criteria, restrictions, and adverse events.

Blood sampling was performed pre-dose and 0.5 h, 1:00 h, 2:00 h, 2:30 h, 3:00 h, 3:30 h, 4:00 h, 4:30 h, 5:00 h, 6:00 h, 8:00 h, 12:00 h post-dose, as well as after 24 h, 36 h, 48 h and 72 h.

There was a wash-out period of 14 days after the first study phase to exclude a pharmacokinetic carry-over effect, before participants entered phase 2 of the study. It was also chosen according to the terminal elimination half-life of drospirenone. After more than 5 half-lives a pharmacokinetic carry-over effect can be excluded [16]. The undertaken procedures were the same as described for phase 1.

### Analytical procedures

The concentration of total (i.e., free, and protein-bound) DRSP was determined using liquid chromatography and double-sector mass spectrometry [LC/MS/MS] in accordance with the respective recommendation for determination of DRSP in PK studies [17].

DRSP was analyzed by the bioanalytical division of Anapharm Europe using the analytical method SOP ANE 5199.05 entitled “Determination of Drospirenone in Human EDTA Plasma over a Concentration Range of 0.25 to 100 ng/mL using a LC/MS/MS Method”. The method involved a solid-phase extraction procedure with reversed phase 60 mg cartridges and subsequent derivatization with Girard-P solution. DRSP and internal standard were measured by reversed phase high-performance liquid chromatography coupled to a tandem mass spectrometry detector (LC/MS/MS). The calibration range at on-line validation was 0.25–99.80 ng/mL. The lowest calibrator (and thus the limit of quantification) was 0.25 ng/mL. The on-line validation based on quality control samples at four concentration levels (0.75, 35.00, 75.00, 8.00 ng/mL) for DRSP measured twice per analytical run showed an inter-assay precision of 2.17–6.72% coefficient of variation (CV). All samples from the same subject were measured in a single analytical run to eliminate the influence of the inter-assay imprecision of the assessment [17–19].

### Pharmacokinetic endpoints

The following pharmacokinetic endpoints were defined for DRSP:

AUC(0-72 h) Area under the concentration/time curve, calculated by the trapezoidal rule from time 0 h to 72 h:

*Cmax*: Observed maximal concentration after administration.

*tmax*: Observed time point of maximal concentration.

The highest concentration really measured and the time at which it was registered in any given volunteer was regarded as Cmax and tmax, respectively. In cases with two or more identical concentration maxima at different time points the first one was always regarded as tmax.

If differences between the planned and real blood sampling times (time deviations) were observed after the administration of the test product the real time intervals were used for the purpose of calculation of the pharmacokinetic endpoints.

In case of missing samples because of not coming to visit or in case of drop-out, all available plasma samples of this subject had to be analysed in the bioanalytical center and the results were to be presented in the study report as concentrations and individual graphics. However, no dropouts and no missing samples were recorded in the present study.

All endpoints listed above were determined in a model-independent way with the program SAS for Windows version 9.2; NC_PKP.sas (Statistical Analysis System, SAS Institute, Cary, NC, USA).

All pharmacokinetic endpoints were determined in a model-independent way. The highest concentration really measured and the time at which it has been registered after each dose in any given volunteer was regarded as Cmax and tmax respectively.

The primary endpoints in the present trial were AUC(0-72 h) and Cmax of DRSP. These endpoints had to undergo descriptive and comparative statistical evaluation.

Secondary endpoint was tmax of DRSP and had to undergo descriptive statistical evaluation.

### Statistics

Descriptive statistical evaluation provided the arithmetic and geometric means, standard deviation, CV, minimum, maximum, and median of the following: safety and demographic data of all randomized subjects, blood concentrations per subject/treatment for all randomized subjects and PK endpoints for all randomized subjects.

We performed the analysis of variance of log-transformed data according to a general linear model (GLM-ANOVA). Fixed factors in the model were sequence, treatment, period and subject within sequence.

We carried out the biostatistical evaluation using SAS for Windows version 9.2 (Statistical Analysis System, SAS-Institute, Cary NC, USA).

For the pharmacokinetic endpoints a descriptive statistical evaluation for all PK endpoints after single dose administration with and without food intake was performed. Aparametric method (ANOVA-log) for the primary endpoints AUC(0-72 h) and Cmax of DRSP was carried out.

A 90% confidence interval (CI) for the ratio (Test under fed vs. Test under fasting conditions) for the primary endpoints AUC(0-72 h) and Cmax of DRSP was used with following fixed factors in the model: sequence, treatment, period, volunteer within sequence. A non-parametric method (Hauschke et al. 1990) for tmax of DRSP was used.

A descriptive statistical evaluation was used for the evaluation of the safety.

We selected the 90% CI in accordance with the Committee for Medicinal Products for Human Use (CHMP) Guideline on the Investigation of Bioequivalence (CPMP/EWP/QWP/1401/98 Rev. 1/Corr**), dated 20 January 2010, stating that in “studies to determine bioequivalence after a single dose, the parameters to be analyzed are AUC(0-t), or, when relevant, AUC_(0-72 h)_ and Cmax, and that for these parameters the 90% CI for the ratio of the test and reference products should be contained within the acceptance interval of 80.00–125.00%. For studies to determine bioequivalence of immediate-release formulations at steady-state, AUC(0-τ) and Cmax, should be analyzed using the same acceptance interval as stated above.” [20]

## Results

### Subject disposition

**Fig. 1:**
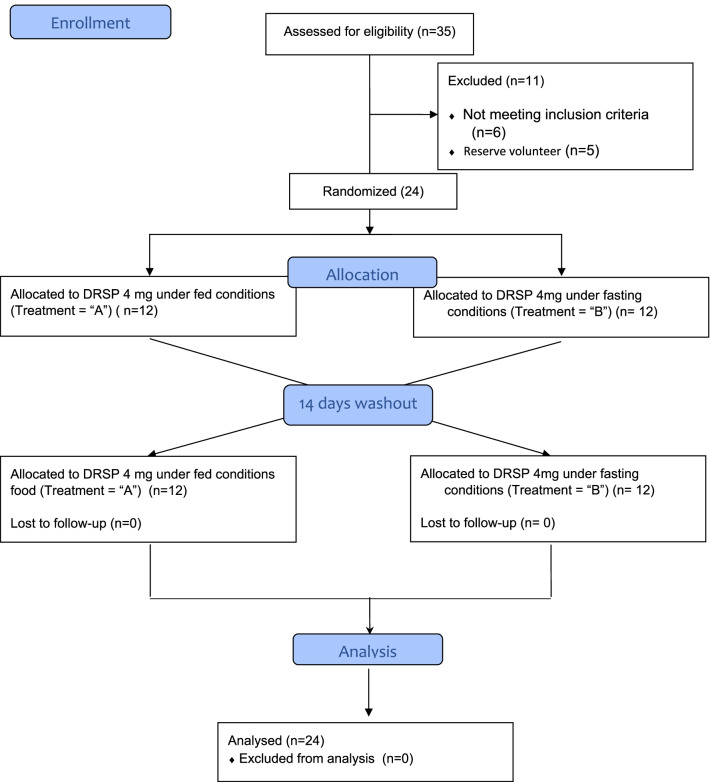
Flow diagram of the patients enrolled in the clinical trial

We questioned a total of 35 pre-menopausal female subjects with respect to the inclusion and exclusion criteria and performed standard clinical and laboratory screening at the entry examination. Eleven of the 35 enrolled subjects were screened but not randomized (see consort flow diagram in Fig. 1). The demographic data of all randomized subjects (*n* = 24) are summarized in Table 1.

**Table 1:** Patient’s characteristics

(*n* = 24)	Mean ± SD	Min–Max
Age [years]	30.5 ± 5.5	19.0–39.0
Height [cm]	161.5 ± 6.2	153.0–172.0
Weight [kg]	62.6 ± 10.8	49.5–87.3
BMI [kg/m^2^]	23.9 ± 3.4	19.2–29.5
male: female	0: 24	

*SD* Standard deviation. *Mi–Max* lowest and highest observed value

The 24 study-completers were exposed to a single oral dose of DRSP 4 mg. The actual wash-out phase between both study periods was 14 days.

#### Pharmacokinetics

A total number of 24 volunteers completed the trial according to the protocol. The samples of 24 study completers were analysed and statistically evaluated.

**Fig. 2:**
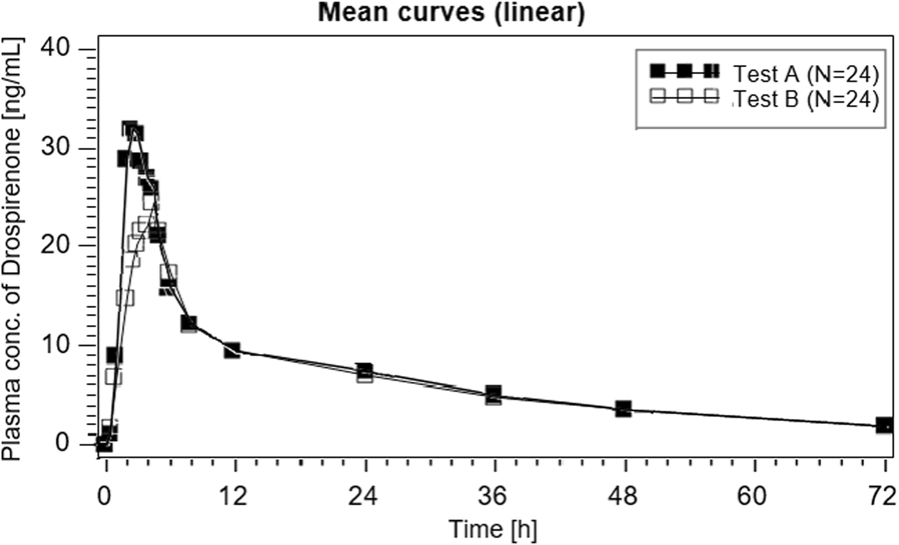
Mean drospirenone plasma concentration–time profile (linear) after single dose of 4 mg drospirenone administered after food intake ‘**A**’ and under fasting conditions ‘**B**’

**Fig. 3:**
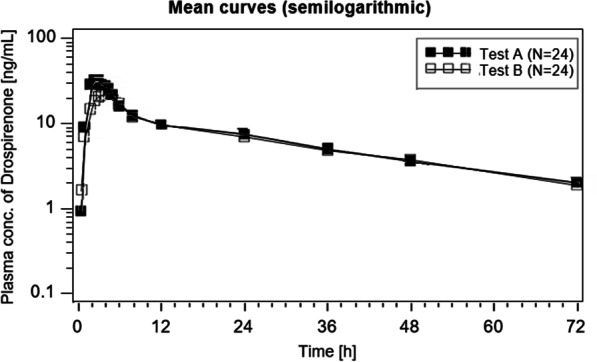
Mean drospirenone plasma concentration–time profile (semilogarithmic) after single dose of 4 mg drospirenone administered after food intake ‘**A**’ and under fasting conditions ‘**B**’

The mean concentration–time curves of DRSP after administration of an oral single dose of 4 mg DRSP under fasting and fed conditions are shown in Figs. 2 and 3 (linear and semilogarithmic).

The evaluation of bioavailability of the endpoints AUC(0-72 h) and Cmax of DRSP was based on a parametric method (ANOVA-log). The 90% CI for the intra-individual ratios (Test with food ‘A’ vs. Test without food ‘B’) for AUC(0-72 h) and Cmax of DRSP are presented in Table 2.

**Table 2:** Pharmacokinetic endpoints of drospirenone after an oral single dose of 4 mg drospirenone administered after food intake and under fasting conditions (geometric mean, arithmetic mean, SD, CV, lower and upper ranges, median, *n* = 24)

Drospirenone							
Variable	Geom mean	Arithm mean	SD	CV	Range	Median	*N*
*TEST ‘A’ (with food)*							
AUC(0-72 h) [ng*h/mL]	479.28	487.93	96.00	19.7	311.46—733.36	462.40	24
Cmax [ng/mL]	34.98	36.19	9.83	27.1	20.60—59.39	35.42	24
tmax [h]	2.698	2.896	1.343	46.4	2.000- 8.000	2.500	24
*TEST ‘B’ (without food)*							
AUC(0-72 h) [ng*h/mL]	443.83	452.38	91.13	20.1	303.76—671.67	438.23	24
Cmax [ng/mL]	27.04	27.53	5.17	18.8	17.99—35.02	28.24	24
tmax [h]	3.944	4.063	0.958	23.6	2.500—6.000	4.500	24

*CV* coefficient of variation. *AUC(0–72)* Area under the concentration/time curve from time 0 h to the last observed concentration at 72 h. *Tmax* timepoint of maximal concentration; *Cmax* observed maximal concentration after administration

The 90% CI calculated by means of ANOVA-log for the endpoint, intra-individual ratio (Test ‘A’ vs. Test ‘B’) of AUC(0-72 h) of DRSP was between 104.72 and 111.36%. The 90% CI calculated by means of ANOVA- log for the endpoint intra-individual ratio (Test ‘A’ vs. Test ‘B’) of Cmax of DRSP was between 118.58 and 141.10% (see Table 3).

**Table 3:** 90% confidence intervals of drospirenone

Drospirenone (*n* = 24)				
Variable	Method	Point estimator	Confidence intervals	CV(%)
AUC(0-72 h) (Ratio TEST ‘A’ with food vs. TEST ‘B’ without food)	ANOVA-log	107.99%	104.72%–111.36%	6.21%
Cmax (Ratio TEST ‘A’ with food vs. TEST ‘B’ without food)	ANOVA-log	129.35%	118.58%–141.10%	17.68%

*CV* coefficient of variation. *AUC(0–72)* Area under the concentration/time curve from time 0 h to the last observed concentration at 72 h. *Tmax* timepoint of maximal concentration; *Cmax* observed maximal concentration after administration

The mean relative bioavailability of the Test with food ‘A’ compared to the Test without food ‘B’ after single dose administration based on the endpoints AUC(0-72 h) was 107.99% and for the endpoint Cmax it was 129.35%

### Adverse events

No serious AEs or unexpected AEs occurred during the study.

## Discussion

The evaluation of the relative bioavailability in this study was based on a parametric method (ANOVA-log) for the pharmacokinetic endpoints AUC(0-72 h) and Cmax of DRSP after intake o food and under fasting conditions. The 90% CI calculated by means of ANOVA-log for the endpoint, intra-individual ratio (Test ‘A’ vs. Test ‘B’) of AUC(0-72 h) of DRSP was between 104.72 and 111.36%. The 90% CI calculated by means of ANOVA-log for the endpoint intra-individual ratio (Test ‘A’ vs. Test ‘B’) of Cmax of DRSP was between 118.58 and 141.10%.The mean relative bioavailability of the Test with food ‘A’ compared to the Test without food ‘B’ after single dose administration based on the endpoints AUC(0–72 h) was 107.99% and for the endpoint Cmax it was 129.35%.The single dose administration of 4 mg non micronized DRSP taken after a standard high-fat breakfast has a relative bioavailability of 107.99% for AUC(0–72 h).

The rate of absorption, based on the endpoint Cmax of DRSP was increased by about 30% under fed conditions which differs from information reported in the US Prescribing Information of YAZ® (tablets containing 3 mg DRSP and 0.02 mg EE). According to the latter, the rate of absorption of DRSP in the combination with EE following single administration of a formulation similar to YAZ® was slower under fed (high fat meal) conditions with the serum Cmax being reduced by about 40% for both components [3].

EE is an inhibitor of CYP3A4 [18–20] and our findings are in line with those previously reported by Kasserra et al. 2015 [21] and Wiesinger et al. 2015 [12]; however, the present study could not determine which metabolic pathway is responsible for the observed increased exposure to DRSP when co-administered with EE. Contradictory in-vitro data leave doubt as to whether CYP3AA inhibition is the main contributor or not.

Considering the metabolic pathway of DRSP, sulfotransferases in addition to CYP3A4 are a possible target for a drug-drug interaction for EE and DRSP. Rohn et al. [22] and Rohn-Glowacki [23] recently explored the potent inhibition by EE of human SULT1A1, the major xenobiotic sulfating isoform in the liver. The isoforms of greatest interest in these studies were SULT1E1, known to sulfate EE at nanomolar levels (Falany et al., 1995, [24], Falany and Falany, 1997 [25]), and SULT1A2, which has the most similar loop 1 amino acid sequence including Ile89 identical to SULT1A1. The inhibition of SULT1E1 sulfation activity by EE would be competitive since it is known to be a substrate.

Possible explanations for the different effect of food on the test product as compared to literature data might be the following:

1. The fact that the data related to YAZ® refer to a combination of DRSP with ethinyl oestradiol. The food effect might be different for the combination as compared to the single component
2. The fact that completely different galenic formulations were investigated. Differences in the formulation might also lead to a difference in the effect of food.
3. Our results may also be due to the non-micronized formulation of the DRSP 4 mg tablets.

The observed differences neither affect inhibition of ovulation [26] nor the clinical efficacy that was demonstrated in 2 European [27] and 1 US clinical trial [28] as we could find similar AUCs for both regimes; the fasten and the non-fasten regime.

The non-existing AUC differences in the absorption of the DRSP only pill between food intake and non-food intake is at least an increase in the safety profile of this non micronized DRSP formulation as constant hormonal levels are garneted independent of the consumers habits in real life.

## Data Availability

All data generated or analysed during this study are included in this published article.

## Abbreviations

DRSP: Drospirenone
AUC: Area under the curve
Cmax: Observed maximal concentration after administration
Tmax: Observed time point of maximal concentration
POP: Progestin only pill
CYP: Cytochrome P
SHBG: Sexual hormone binding globuline
CBG: Corticosteroid-binding globulin
AR: Androgen receptor
PK: Pharmakokinetics
COC: Combined oral contraceptive
EE: Ethinyl estradiol
LC/MS/MS: Liquid chromatography coupled to a tandem mass spectrometry detector
SULT1A1: Sulfotransferase 1A1
CV: Coefficient of variation (relative standard deviation/mean × 100)
